# Two new species of the genus *Betacixius* Matsumura, 1914 from China (Hemiptera, Fulgoromorpha, Cixiidae)

**DOI:** 10.3897/zookeys.956.50195

**Published:** 2020-08-06

**Authors:** Yan Zhi, Chang-Hua Zhang, Lin Yang, Xiang-Sheng Chen

**Affiliations:** 1 Institute of Entomology, Guizhou University, Guiyang, Guizhou, 550025, China Guizhou University Guiyang China; 2 Laboratory Animal Center, Guizhou Medical University, Guiyang, Guizhou, 550025, China Guizhou Tobacco Company Zunyi Branch Zunyi China; 3 The Provincial Special Key Laboratory for Development and Utilization of Insect Resources of Guizhou, Guizhou University, Guiyang, Guizhou, 550025, China Guizhou University Guiyang China; 4 Guizhou Tobacco Company Zunyi Branch, Zunyi, Guizhou, 563000, China Guizhou Tobacco Company Zunyi Branch Zunyi China

**Keywords:** Female genitalia, Fulgoroidea, morphology, Oriental region, taxonomy

## Abstract

Two new species of *Betacixius* Matsumura, 1914 (Fulgoromorpha, Cixiidae), *B. latissimus* Zhi & Chen, **sp. nov.** and *B. maguanensis* Zhi & Chen, **sp. nov.**, are described from Yunnan Province, China. The genus is reviewed and a key to all known species is provided. The females of four Chinese species are described for the first time.

## Introduction

*Betacixius* is a small genus established by Matsumura (1914) for 23 species in the subfamily Cixiinae (Hemiptera, Fulgoromorpha, Cixiidae), with *B. ocellatus* Matsumura, 1914 as the type species. *Betacixius* belongs to the tribe Semonini (Emeljanov 2002) and has been taxonomically studied by various authors, with a number of species published successively (e.g. Jacobi 1944; Fennah 1956; Hori 1982; Tsaur et al. 1991; Zhang and Chen 2011).

Recent study of some Chinese specimens has found two new species, *B. latissimus* Zhi & Chen, sp. nov. and *B. maguanensis* Zhi & Chen, sp. nov., which are described here. Female specimens of four species are also described for the first time. So far, including the two new species, the genus currently now counts for 25 valid species and two subspecies, all distributed in the Palaearctic and Oriental regions (Bourgoin 2020).

## Materials and methods

The morphological terminology and measurements follow Bourgoin (1987) and Bourgoin et al. (2015). The morphological terminology of female genitalia follows Bourgoin (1993). Body length was measured from apex of vertex to tip of forewing; vertex length was measured the median length of vertex (from apical transverse carina to tip of basal emargination). Fuchsin staining was used to highlight female genitalia structures studied. External morphology and drawings were made with the aid of a Leica MZ 12.5 stereomicroscope. Photographs were taken with KEYENCE VHX-1000 system. Illustrations were scanned with a CanoScan LiDE 200 flatbed scanner and imported into Adobe Photoshop 7.0 for labeling and figure composition. The distribution map was generated with Google Earth Pro v. 7.3.2 (Google LLC). The dissected male and female genitalia are preserved in glycerin in small plastic tubes pinned together with the specimens.

The type specimens are deposited in the Institute of Entomology, Guizhou University, Guiyang, Guizhou Province, China (**GUGC**).

## Taxonomy

### Betacixius

Taxon classificationAnimaliaHemipteraCixiidae

Matsumura, 1914

68D17DBD-2D60-5455-999D-CCA32662E2A3

Betacixius Matsumura, 1914: 412; Tsaur et al. 1991: 27; Zhang and Chen 2011.

#### Type species.

*Betacixius ocellatus* Matsumura, 1914, by original designation.

#### Diagnosis.

See Zhang and Chen (2011: 48).

#### Distribution.

China, Japan, Vietnam.

### Key to species of *Betacixius*^1^

**Table:** 

1	Forewing with markings	**2**
–	Forewing without any markings	**21**
2	Forewing with a large ocellate marking in apical half	**3**
–	Forewing without ocellate marking in apical half	**6**
3	Forewing with an oblique, brown band extending from clavus across middle of corium	***B. tonkinensis* Matsumura, 1914**
–	Forewing without such a band	**4**
4	Endosoma of aedeagus with one spine, hook-shaped (Zhang and Chen 2011: figs 22, 23)	***B. flagellihamus* Zhang & Chen, 2011**
–	Endosoma of aedeagus with two spines, not hook-shaped	**5**
5	Periandrium of aedeagus apically with two L-shaped processes	***B. maculosus* Tsaur & Hsu, 1991**
–	Periandrium of aedeagus apically with one nearly straight and one arched processes	***B. ocellatus* Matsumura, 1914**
6	Forewing with an oblique band extending from stigma passing through its middle part	**7**
–	Forewing without such a band	**13**
7	Forewing with apical cells of M and Cu strongly infuscate	***B. transversus* Jacobi, 1944**
–	Forewing with apical cells not infuscate	**8**
8	Forewing with apical margin black or distinctly darkened	**9**
–	Forewing with apical margin fuscous or not distinctly darkened	**10**
9	Frons with a pallid spot at centre of lateral margins; mesonotum testaceous	***B. kumejimae* Matsumura, 1914**
–	Frons without such spots; mesonotum, except scutellum, castaneous-piceous	***B. euterpe* Fennah, 1956**
10	Forewing with a spot near sutural margin of clavus near union of claval veins, no oblique dark band at this level extending into corium	**11**
–	Forewing with an oblique dark band extending from clavus into centre of corium, slightly distad of level of union of claval veins	**12**
11	Forewing basally with a broad transverse band from dorsal margin to sutural margin of clavus (Fig. 3E)	***B. latissimus* sp. nov.**
–	Forewing without above band	***B. obliquus* Matsumura, 1914**
12	Forewing basally with a light brown band	***B. pallidior* Jacobi, 1944**
–	Forewing basally without band	***B. michioi* Hori, 1982**
13	Forewing with a long black stripe from base, along clavus extending to Rs	***B. fuscus* Tsaur & Hsu, 1991**
–	Forewing without such a stripe	**14**
14	Forewing along the R with a black stripe widened towards the rear	***B. robustus* Jacobi, 1944**
–	Forewing without such a stripe	**15**
15	Anal segment asymmetrical	***B. nelides* Fennah, 1956**
–	Anal segment symmetrical	**16**
16	Ventral margin of periandrium basally with two broad, lobate processes (Zhang and Chen 2011: figs 10, 11)	***B. bispinus* Zhang & Chen, 2011**
–	Ventral margin of periandrium basally without process	**17**
17	Endosoma apically without spinose process	**18**
–	Endosoma apically with one or two spinose processes	**19**
18	Spinose process on right side of periandrium medium-sized, curved upwards, apex dorsally directed; spinose process on left side parallel to periandrium for most potion, apex ventrocephalically directed	***B. rinkihonis* Matsumura, 1914**
–	Spinose process on right side of periandrium very short, nearly straight, apex directed cephalad; spinose process on left side generally dorsocephalically directed	***B. shirozui* Hori, 1982**
19	In lateral view, apical lobe of anal segment ventrally rounded	***B. delicatus*Tsaur & Hsu, 1991**
–	In lateral view, apical lobe of anal segment ventrally pointed	**20**
20	Spinose process on right side of periandrium near dorsal margin, coiled 90 degrees to left; endosoma with two spinose processes	***B. sparsus* Tsaur & Hsu, 1991**
–	Spinose process on right side of periandrium near ventral margin, nearly straight, apex directed cephalad; endosoma with one spinose process	***B. maguanensis* sp. nov.**
21	Endosoma of aedeagus apically with two processes	**22**
–	Endosoma of aedeagus apically with one process	**23**
22	Ventral margin of periandrium with a long process	***B. flavovittatus* Hori, 1982**
–	Ventral margin of periandrium without process	***B. nigromarginalis* Fennah, 1956**
23	Frons without median carina	***B. clypealis* Matsumura, 1914**
–	Frons with median carina	**24**
24	Body pale brown; periandrium of aedeagus with two processes on right side	***B. brunneus* Matsumura, 1914**
–	Body green; periandrium of aedeagus with one process on each side	***B. herbaceus* Tsaur & Hsu, 1991**

### Betacixius bispinus

Taxon classificationAnimaliaHemipteraCixiidae

Zhang & Chen, 2011

C7A948EB-9D60-5F97-9306-127BD2F38468

Figure 1A–I

Betacixius bispinus Zhang & Chen, 2011: 53, figs 1–13, 24.

#### Material examined.

**China**: 1♂, Guizhou Province, Yanhe County, Mayanghe National Natural Reserve (600 m) (28°40'N, 108°19'E), 5–12 June 2007, Xiang-Sheng Chen (holotype); 1♂ 7♀♀, Guizhou Province, Yanhe County, Mayanghe National Natural Reserve (600 m), 5–12 June 2007, Xiang-Sheng Chen (paratypes); 1♂ 1♀, Guizhou Province, Tongren City, Fanjingshan National Natural Reserve (500–1800 m) (27°54'N, 108°38'E), 1–3 June 2002, Xiang-Sheng Chen; 2♂♂ 1♀, Guizhou Province, Daozhen County, Sanqiao Town (29°3'N, 107°30'E), 24 May 2002, Xiang-Sheng Chen; 1♂, Guizhou Province, Chishui City, Shizhangdong Waterfall (28°22'N, 105°43'E), 30 May 2006, Zai-Hua Yang; 27♂♂ 25♀♀, Guizhou Province, Suiyang County, Kuankuoshui National Natural Reserve (28°14'N, 107°12'E), 8–9 June 2010, Pei Zhang, Bin Zhang, Yu-Jian Li, Ji-Chun Xing; 2♂♂ 1♀, Guizhou Province, Huishui County, Duanshan Town, Guangrong Village (25°50'N, 106°37'E), 9 May 2013, Jian-Kun Long, Zai-Hua Yang; 1♂ 1♀, Sichuan Province, Nanchong City, Dayou Township (30°48'N, 106°41'E), 9–10 May 2008, Zheng-Guang Zhang, Zai-Hua Yang; 2♂♂ 2♀♀, Yunnan Province, Longling County (24°35'N, 98°41'E), 23 May 2016, Meng-Shu Dong; 4♂♂ 3♀♀, Yunnan Province, Maguan County, Gulinqing Township (22°48'N, 103°57'E), 30–31 May 2016, Liang-Jing Yang, Qiang Luo, Ying-Jian Wang.

#### Supplementary description.

**Figure 1.**
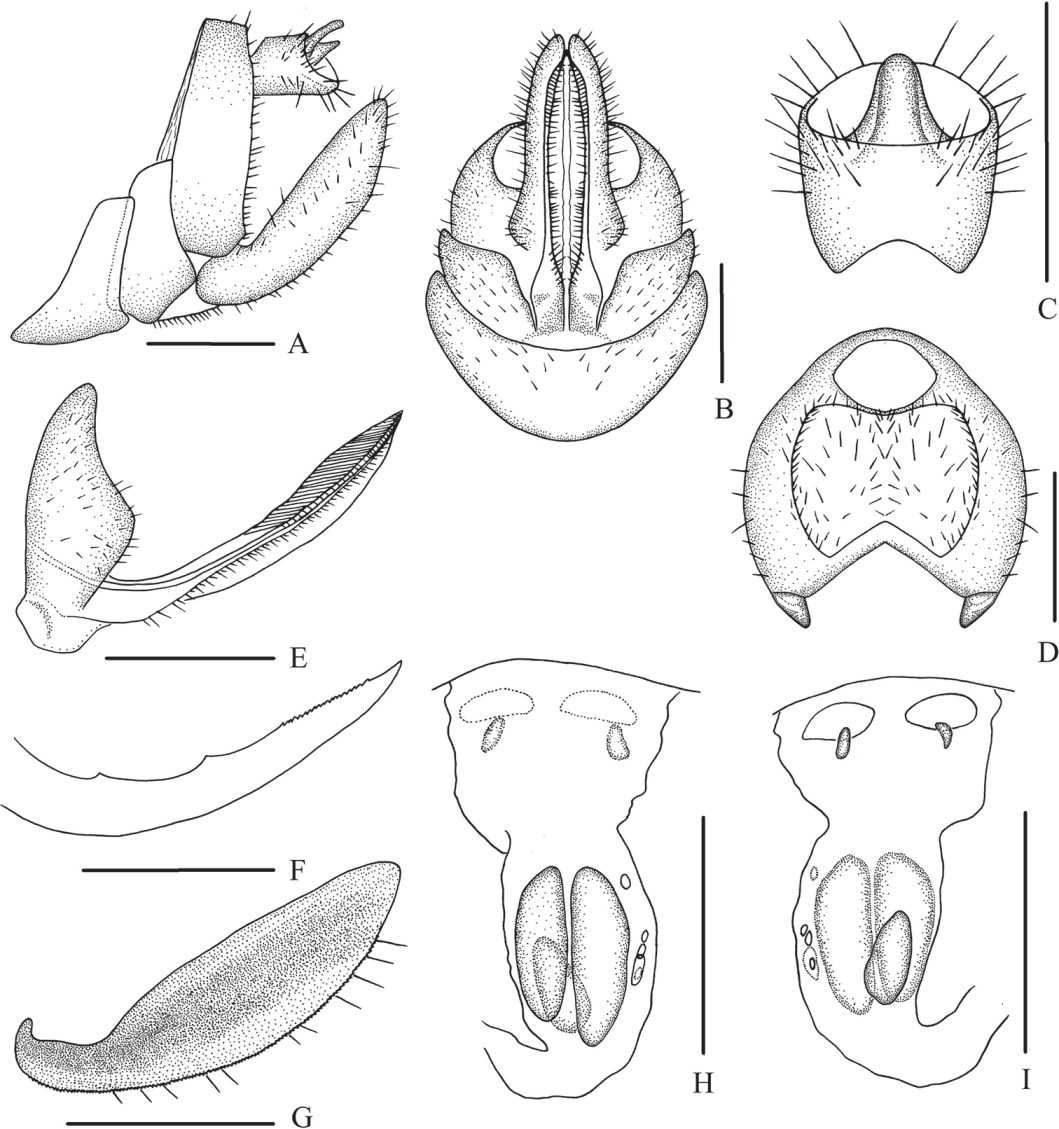
*Betacixius bispinus* Zhang & Chen, 2011, female **A** genitalia, lateral view **B** genitalia, ventral view **C** anal segment, dorsal view **D** tergite IX, caudal view **E** gonapophysis VIII and gonocoxa VIII, ventral view **F** gonapophysis IX, lateral view **G** gonoplac, inner lateral view **H** posterior vagina, ventral view **I** posterior vagina, dorsal view. Scale bars: 0.5 mm.

Female genitalia. Tergite IX (Fig. 1A, B, D) moderately sclerotized, with a large nearly dumbbell-shaped wax plate. Anal segment (Fig. 1C) rectangular, widening to apex, 1.1 times wider than long in dorsal view, anal style strap-like. Gonapophysis VIII (Fig. 1E) elongate, and slightly curved upwards. Gonapophysis IX (Fig. 1F) with two middle teeth, distance ratio between distal middle tooth to apex and length of denticulate portion is 2.2. Gonoplac (Fig. 1G) rod-like, 3.5 times longer than wide in lateral view. Posterior vagina pattern as shown in Figure 1H, I.

#### Distribution.

China (Guizhou, Sichuan, Yunnan).

#### Note.

The female genitalia of this species are described and illustrated for the first time.

### Betacixius flagellihamus

Taxon classificationAnimaliaHemipteraCixiidae

Zhang & Chen, 2011

22DE5F3B-9328-570D-AB1A-9D9484EB1111

Figure 2A–I

Betacixius flagellihamus Zhang & Chen, 2011: 54, figs 14–23, 25.

#### Material examined.

**China**: 1♂, Guizhou Province, Leishan County, Leigongshan National Natural Reserve (26°31'N, 108°17'E), 13 May 1985, Zi-Zhong Li (holotype); 7♂♂ 9♀♀, Guizhou Province, Leishan County, Leigongshan National Natural Reserve, 13 May 1985, Zi-Zhong Li (paratypes); 2♂♂, Guizhou Province, Guiyang City, June 1983, Students of Grade 79, Major Plant Protection (paratypes); 1♂ 2♀♀, Guizhou Province, Guiyang City, Forest Park (1000 m) (26°32'N, 106°45'E), 20 May 2007, Xiang-Sheng Chen (paratypes); 1♂ 1♀, Guizhou Province, Guiyang City, Forest Park, 14–17 May 1984, collector unknown; 2♂♂ 1♀, Guizhou Province, Duyun City, Gantang Town, Tuanzhai Village (26°16'N, 107°26'E), 12 May 2014, Ming Ning, Gai-Ping Yang; 1♂5♀♀, Guizhou Province, Suiyang County, Kuankuoshui National Natural Reserve (28°14'N, 107°12'E), 8–9 June 2010, Pei Zhang, Bin Zhang; 3♂♂ 2♀♀, Guizhou Province, Huishui County, Duanshan Town, Guangrong Village (25°50'N, 106°37'E), 9 May 2013, Jian-Kun Long, Zai-Hua Yang; 1♂ 3♀♀, Guizhou Province, Guiyang City, Huaxi District (26°25'N, 106°40'E), 15–16 May 1982, collector unknown; 2♂♂ 3♀♀, Guizhou Province, Guiyang City, Huaxi District, 1 May 2008, Zheng-Guang Zhang; 1♂, Guizhou Province, Libo County, Maolan Town (25°23'N, 108°4'E), 9 April 2011, Jian-Kun Long; 2♂♂1♀, Guizhou Province, Guiyang City, Forest Park, 24 May 2006, Zai-Hua Yang; 3♂♂ 2♀♀, Guizhou Province, Guiyang City, Forest Park, 21 April 2010, Jun-Qiang Ni; 10♂♂ 6♀♀, Guizhou Province, Guiyang City, Forest Park, 18 May 2012, Wei-Bin Zheng, Yong-Gang Xiao; 1♂, Guizhou Province, Guiyang City, Huaxi District, 29 April 2017, Yong-Jin Sui.

#### Supplementary description.

**Figure 2.**
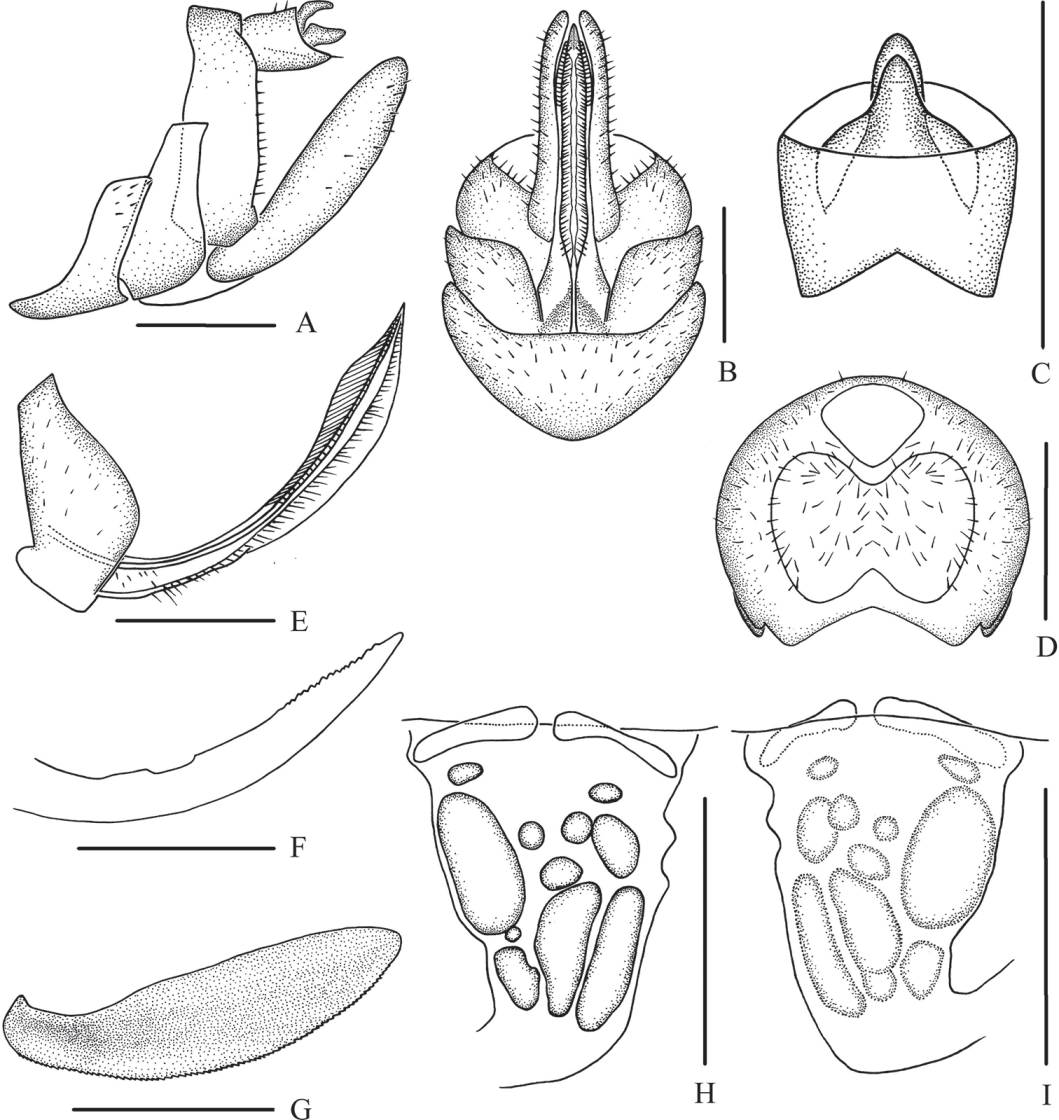
*Betacixius flagellihamus* Zhang & Chen, 2011, female **A** genitalia, lateral view **B** genitalia, ventral view **C** anal segment, dorsal view **D** tergite IX, caudal view **E** gonapophysis VIII and gonocoxa VIII, ventral view **F** gonapophysis IX, lateral view **G** gonoplac, inner lateral view **H** posterior vagina, ventral view **I** posterior vagina, dorsal view. Scale bars: 0.5 mm.

*Female genitalia*. Tergite IX (Fig. 2A, B, D) moderately sclerotized, with a large nearly dumbbell-shaped wax plate. Anal segment (Fig. 2C) rectangular, widening to apex, 1.5 times wider than long in dorsal view, anal style strap-like. Gonapophysis VIII (Fig. 2E) elongate, and slightly curved upwards. Gonapophysis IX (Fig. 2F) with two middle teeth, distance ratio between distal middle tooth to apex and length of denticulate portion is 2.1. Gonoplac (Fig. 2G) rod-like, 3.6 times longer than wide in lateral view. Posterior vagina pattern as shown in Figure 2H, I.

#### Host plant.

*Quercus* sp. (Fagaceae).

#### Distribution.

China (Guizhou).

#### Note.

The female genitalia of this species are described and illustrated for the first time.

### Betacixius latissimus sp. nov.

Taxon classificationAnimaliaHemipteraCixiidae

1A09423C-7E58-51F0-96DD-8D6B5E3B34C3

http://zoobank.org/ED00F996-CE3B-4BAB-8B3A-C3427C482BC5

Figures 3A–M , 4A–I

#### Type material.

***Holotype***: ♂, **China**: Yunnan Province, Jinping County, Fenshuiling National Nature Reserve (22°46'N, 103°13'E), 7–8 June 2016, Liang-Jing Yang, Ying-Jian Wang; paratypes: 1♂ 1♀, same data as holotype.

#### Description.

***Body length***: male 6.9–7.1 mm (*n* = 2), female 7.2 mm (*n* = 1).

**Figure 3.**
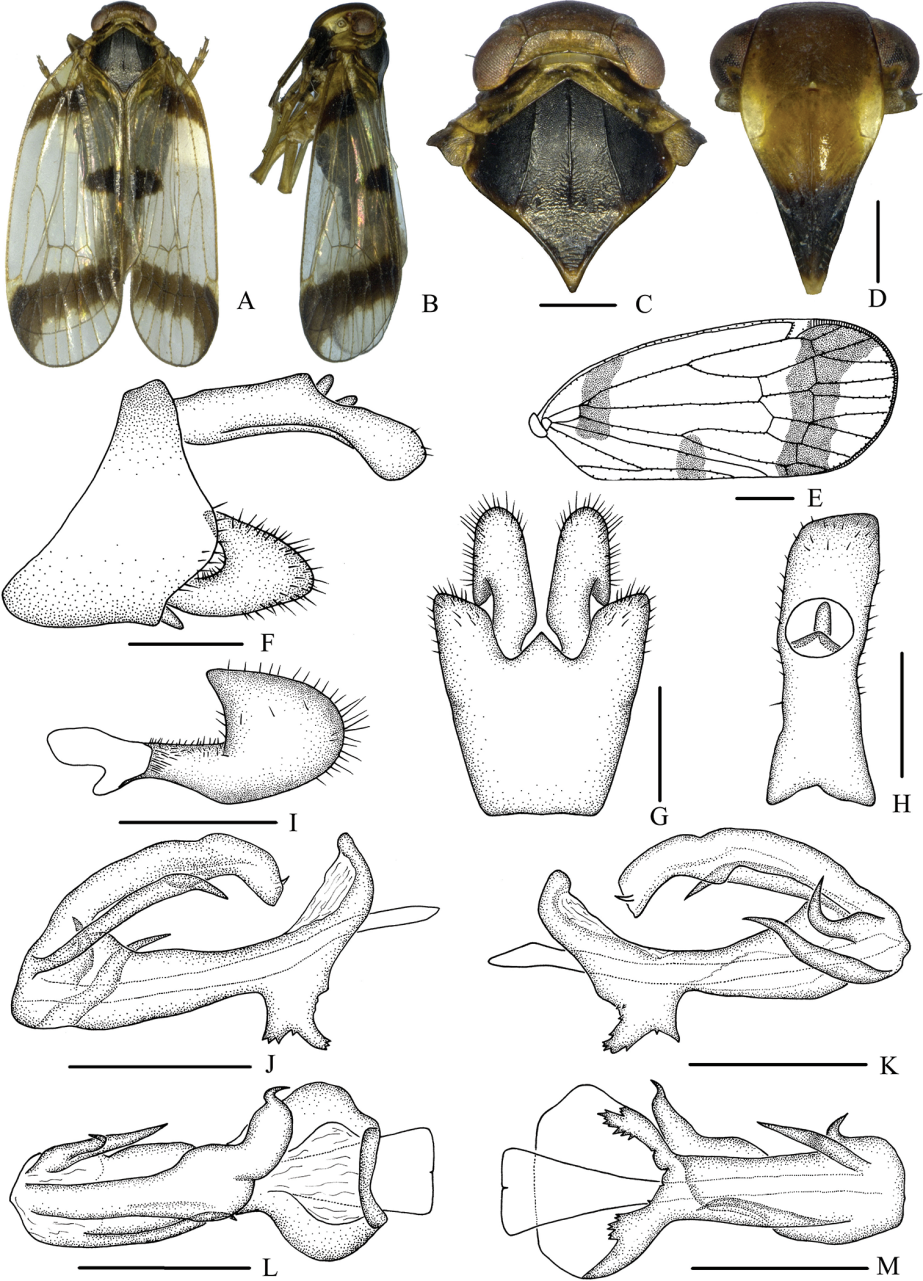
*Betacixius latissimus* sp. nov., male **A** habitus, dorsal view **B** habitus, lateral view **C** head and thorax, dorsal view **D** face, ventral view **E** forewing **F** genitalia, lateral view **G** pygofer and gonostyli, ventral view **H** anal segment, dorsal view **I** gonostyli, inner lateral view **J** aedeagus, right side **K** aedeagus, left side **L** aedeagus, dorsal view **M** aedeagus, ventral view. Scale bars: 0.5 mm (**C,D, F–M**); 1.0 mm (**E**).

***Coloration*.** General color blackish brown (Fig. 3A–D). Eyes reddish brown, lateral ocelli dark red and median ocellus yellow. Vertex dark brown, pronotum yellowish to blackish brown and mesonotum blackish brown. Face generally dark brown, yellowish white above frontoclypeal suture. Postclypeus yellowish brown and anteclypeus blackish brown. Rostrum generally yellowish brown except darker tip. Forewing semi-translucent, base with a broad transverse band from dorsal margin to sutural margin of clavus; clavus with a blackish brown spot on apical third, an oblique transverse band originating from stigma extending to ventral margin. Hind tibiae yellowish brown and abdominal sternites blackish brown.

***Head and thorax*.** Vertex (Fig. 3A, C) broad, 1.9 times wider than long; anterior margin arched convex, posterior margin slightly arched concave; median carina distinct and complete. Frons (Fig. 3D) 0.6 times as long as wide, median carina indistinct, extending from slightly above level of lateral ocelli to median ocellus. Clypeus with median carina distinct and elevated throughout. Pronotum (Fig. 3C) 1.4 times longer than vertex, posterior margin concaved in obtuse angle. Mesonotum 1.6 times longer than pronotum and vertex combined. Forewing (Fig. 3E) 1.9 times longer than wide, with nine apical and five subapical cells; fork Sc+RP slightly distad of fork CuA1+CuA2; first crossvein r-m slightly distad of fork MP; RP 2 branches, MP with four terminals: MP 1, MP2, MP3, and MP4, fork MP1+MP2 almost at same level as fork MP3+MP4. Hind tibia with three lateral spines, six apical spines; chaetotaxy of hind tarsi: 7/7, second segment of hind tarsus with three platellae.

***Male genitalia*.** Pygofer (Fig. 3F, G) symmetrical, dorsal margin concave and U-shaped ventrally, widened towards apex; in lateral view, lateral lobes arched extended caudally. Medioventral process triangular in ventral view. Anal segment (Fig. 3F, H) long, tubular, asymmetrical, apical lobes arched extended ventrally in lateral view, left lobe larger than right one, 2.8 times longer than wide in dorsal view; anal style finger-like, not beyond anal segment. Gonostyli (Fig. 3F, G, I) symmetrical in ventral view; in inner lateral view, apical part extended, apical margin acute. Aedeagus (Fig. 3J–M) in total with four processes. Right side of periandrium with a long spinose process apically, straight for most portion, directed dorsocephalically, apex curved downwards and directed ventrocephalically; left side of periandrium apically with two medium-sized spinose processes, the upper one strongly curved and directed dorsally and the other one slightly curved and directed cephalad. Base of periandrium ventrally with several saw-like teeth. Endosoma slender, structure simple, apically with a small spinose process on left margin.

**Figure 4.**
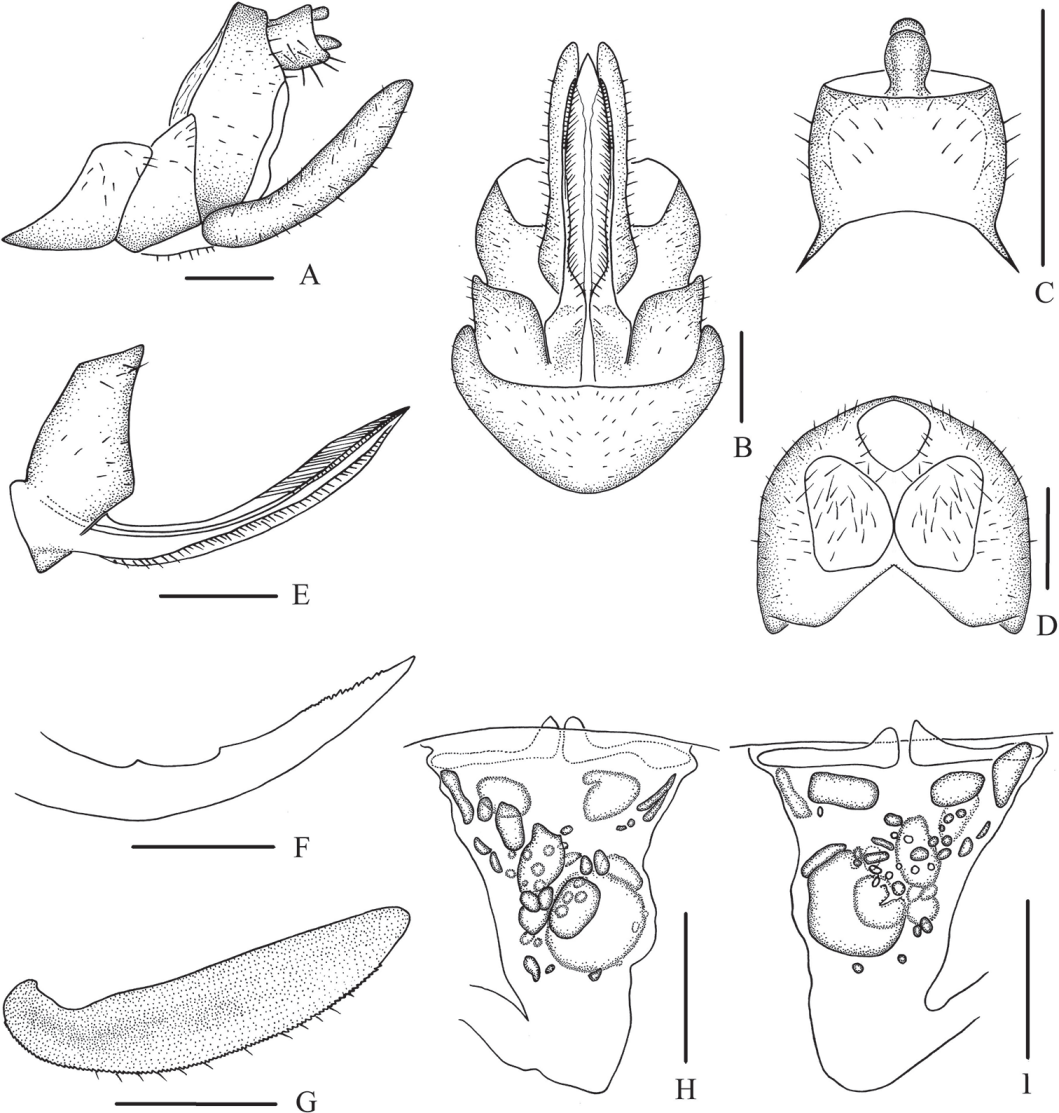
*Betacixius latissimus* sp. nov., female **A** genitalia, lateral view **B** genitalia, ventral view **C** anal segment, dorsal view **D** tergite IX, caudal view **E** gonapophysis VIII and gonocoxa VIII, ventral view **F** gonapophysis IX, lateral view **G** gonoplac, inner lateral view **H** posterior vagina, ventral view **I** posterior vagina, dorsal view. Scale bars: 0.5 mm.

***Female genitalia.*** Tergite IX (Fig. 4A, B, D) moderately sclerotized, with two connected wax plates, nearly oval. Anal segment (Fig. 4C) rectangular, 1.4 times wider than long in dorsal view, anal style strap-like. Gonapophysis VIII (Fig. 4E) elongate, and slightly curved upwards. Gonapophysis IX (Fig. 4F) with two middle teeth, distance ratio between distal middle tooth to apex and length of denticulate portion is 2.4. Gonoplac (Fig. 4G) rod-like, 3.8 times longer than wide in lateral view. Posterior vagina pattern as shown in Figure 4H, I.

#### Distribution.

China (Yunnan).

#### Etymology.

The specific name, derived from Latin word meaning the broadest, refers to the forewing with an extremely broad band at apex.

#### Remarks.

Male genitalia of *B. latissimus* sp. nov. is similar to *B. herbaceus* Tsaur & Hsu, 1991, but differs in: (1) left side of periandrium with two spinose processes (in *B. herbaceus*, left side of periandrium with one spinose process); (2) spinose process on right of periandrium curved downwards (in *B. herbaceus*, spinose process in the same position curved upwards); (3) anal segment asymmetrical (the latter symmetrical); (4) forewing with one blackish brown spot and two bands (the latter without any marking).

### Betacixius maculosus

Taxon classificationAnimaliaHemipteraCixiidae

Tsaur & Hsu, 1991

5596A73B-F298-55AC-88D4-031D5BADD6C6

Figure 5A–I

Betacixius maculosus Tsaur and Hsu in Tsaur et al. 1991: 31, fig. 15A–I.

#### Material examined.

**China**: 3♂♂ 1♀, Taiwan Province, Nantou County (24°2'N, 121°7'E), 25 November 2002, Xiang-Sheng Chen.

#### Supplementary description.

**Figure 5.**
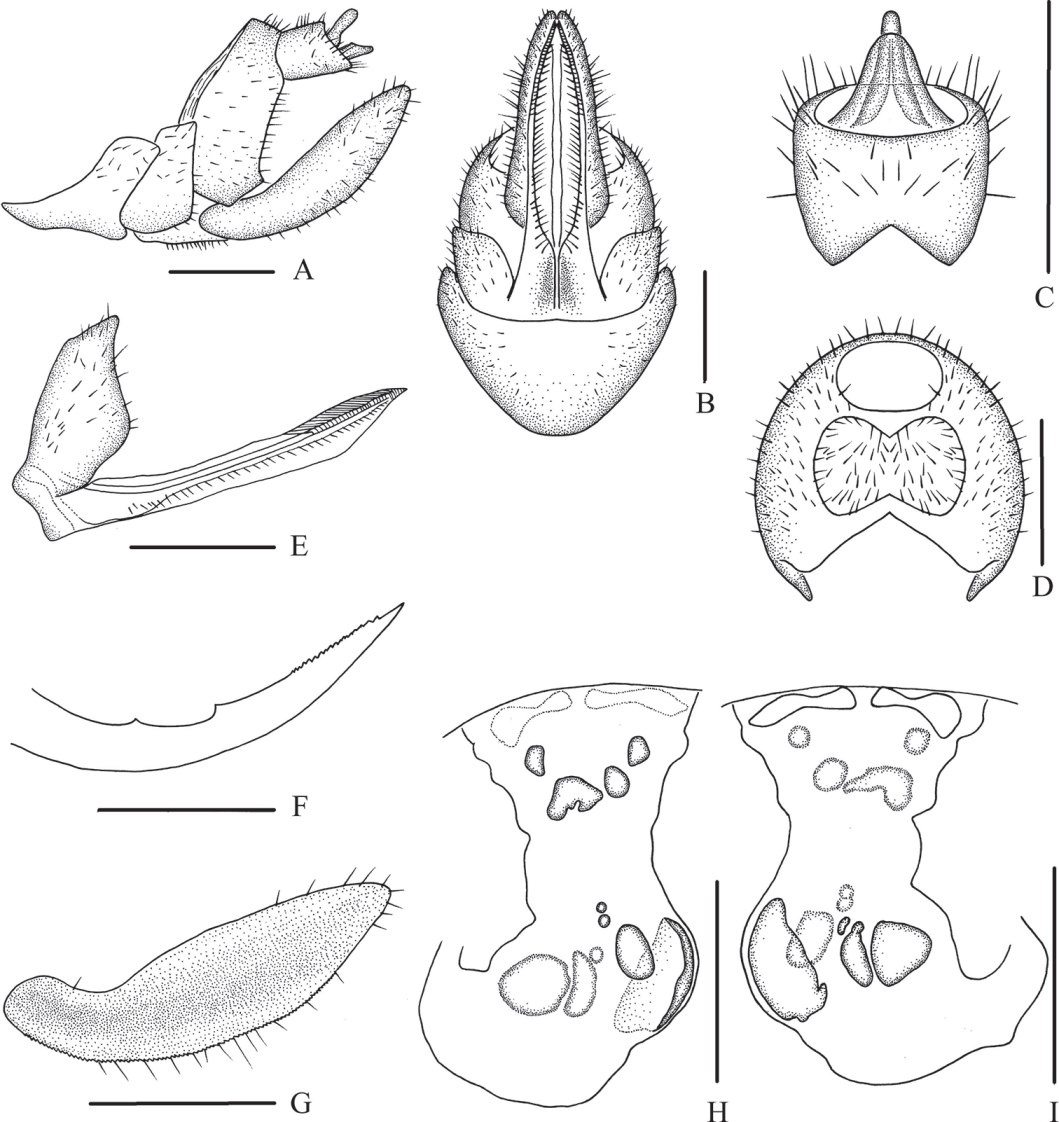
*Betacixius maculosus* Tsaur & Hsu, 1991, female **A** genitalia, lateral view **B** genitalia, ventral view **C** anal segment, dorsal view **D** tergite IX, caudal view **E** gonapophysis VIII and gonocoxa VIII, ventral view **F** gonapophysis IX, lateral view **G** gonoplac, inner lateral view **H** posterior vagina, ventral view **I** posterior vagina, dorsal view. Scale bars: 0.5 mm.

***Female genitalia*.** Tergite IX (Fig. 5A, B, D) moderately sclerotized, with a medium-sized, nearly dumbbell-shaped wax plate. Anal segment (Fig. 5C) rectangular, slightly widening to apex, 1.1 times longer than wide in dorsal view, anal style finger-like. Gonapophysis VIII (Fig. 5E) elongate, and slightly curved upwards. Gonapophysis IX (Fig. 5F) with two middle teeth, distance ratio between distal middle tooth to apex and length of denticulate portion is 2.1. Gonoplac (Fig. 5G) rod-like, 3.4 times longer than wide in lateral view. Posterior vagina pattern as shown in Figure 5H, I.

#### Distribution.

China (Taiwan).

#### Note.

The female genitalia of this species are described and illustrated for the first time in detail.

### Betacixius maguanensis sp. nov.

Taxon classificationAnimaliaHemipteraCixiidae

BD589887-E040-5989-AFCC-D56A6C52AF9C

http://zoobank.org/2FF88233-4AAE-4F3E-9351-C5759FC12F4E

Figures 6A–M , 7A–I

#### Type material.

***Holotype***: ♂, **China**: Yunnan Province, Maguan County, Gulinqing Township (22°48'N, 103°57'E), 30–31 May 2016, Liang-Jing Yang, Ying-Jian Wang, Qiang Luo; paratypes: 8♂♂ 5♀♀, same data as holotype.

#### Description.

Body length: male 5.9–6.5 mm (*n* = 9), female 6.7–7.0 mm (*n* = 5).

**Figure 6.**
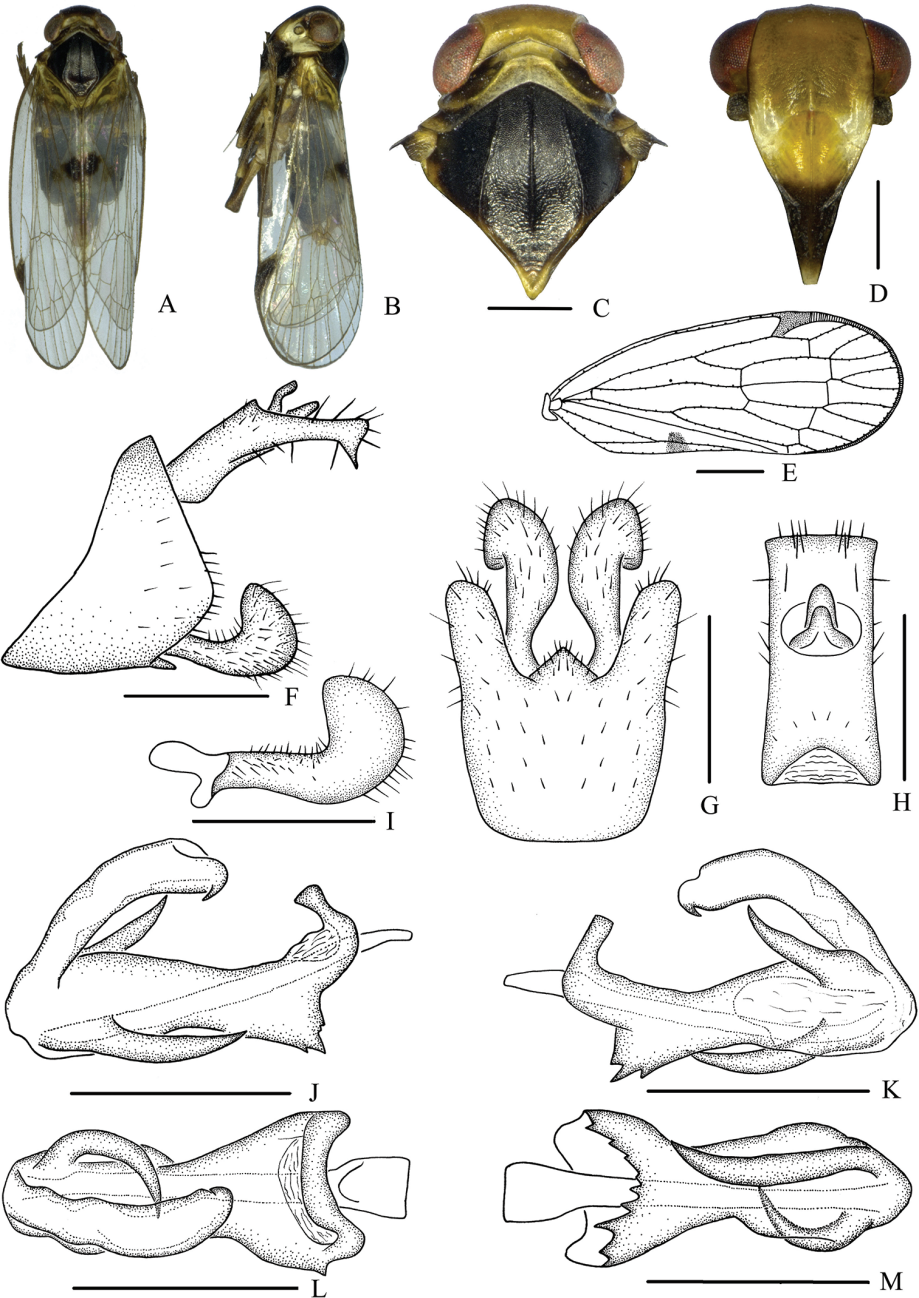
*Betacixius maguanensis* sp. nov., male **A** habitus, dorsal view **B** habitus, lateral view **C** head and thorax, dorsal view **D** face, ventral view **E** forewing **F** genitalia, lateral view **G** pygofer and gonostyli, ventral view **H** anal segment, dorsal view **I** gonostyli, inner lateral view **J** aedeagus, right side **K** aedeagus, left side **L** aedeagus, dorsal view **M** aedeagus, ventral view. Scale bars: 0.5 mm (**C, D, F–M**); 1.0 mm (**E**).

***Coloration*.** General color blackish brown (Fig. 6A–D). Eyes reddish brown, ocelli light yellow. Vertex yellowish brown, pronotum yellowish brown and mesonotum black. Face generally yellowish brown, yellowish white above frontoclypeal suture. Postclypeus yellow and anteclypeus blackish brown. Rostrum generally yellowish brown. Forewing semi-translucent, clavus with a blackish brown spot on apical third, stigma blackish brown. Hind tibiae light blackish brown and abdominal sternites blackish brown.

***Head and thorax*.** Vertex (Fig. 6A, C) broad, 2.6 times wider than long; anterior margin slightly arched convex, posterior margin arched concave; median carina distinct and complete. Frons (Fig. 6D) 0.8 times as long as wide, median carina indistinct, extending from slightly above level of lateral ocelli to median ocellus. Clypeus with median carina distinct and elevated throughout. Pronotum (Fig. 6C) 2.3 times longer than vertex, posterior margin concaved in an obtuse angle. Mesonotum 1.7 times longer than pronotum and vertex combined. Forewing (Fig. 6E) 2.5 times longer than wide, with nine apical and five subapical cells; fork Sc+RP distad of fork CuA1+CuA2; first crossvein r-m almost at same level as fork MP; RP 2 branches, MP with four terminals: MP 1, MP2, MP3, and MP4, fork MP1+MP2 distad of fork MP3+MP4. Hind tibia with three lateral spines, six apical spines; chaetotaxy of hind tarsi: 7/7, second segment of hind tarsus with three platellae.

***Male genitalia*.** Pygofer (Fig. 6F, G) symmetrical, dorsal margin concave and U-shaped ventrally, widened towards apex; in lateral view, lateral lobes triangularly extended caudally. Medioventral process triangular in ventral view. Anal segment (Fig. 6F, H) long tubular, symmetrical, apical lobes ventrally pointed, 2.1 times longer than wide in dorsal view; anal style strap-like, not extending beyond anal segment. Gonostyli (Fig. 6F, G, I) symmetrical in ventral view; in inner lateral view, apical part extended, apical margin rounded. Aedeagus (Fig. 6J–M) with three processes. Right side near ventral margin of periandrium apically with a long spinose process, nearly straight, apex directed cephalad; left side near dorsal margin of periandrium with a shorter spinose process curving upwards, apex right-dorsally directed. Base of periandrium ventrally with several saw-like teeth. Endosoma slender, structure simple, apically with a small hook-like spinose process.

**Figure 7.**
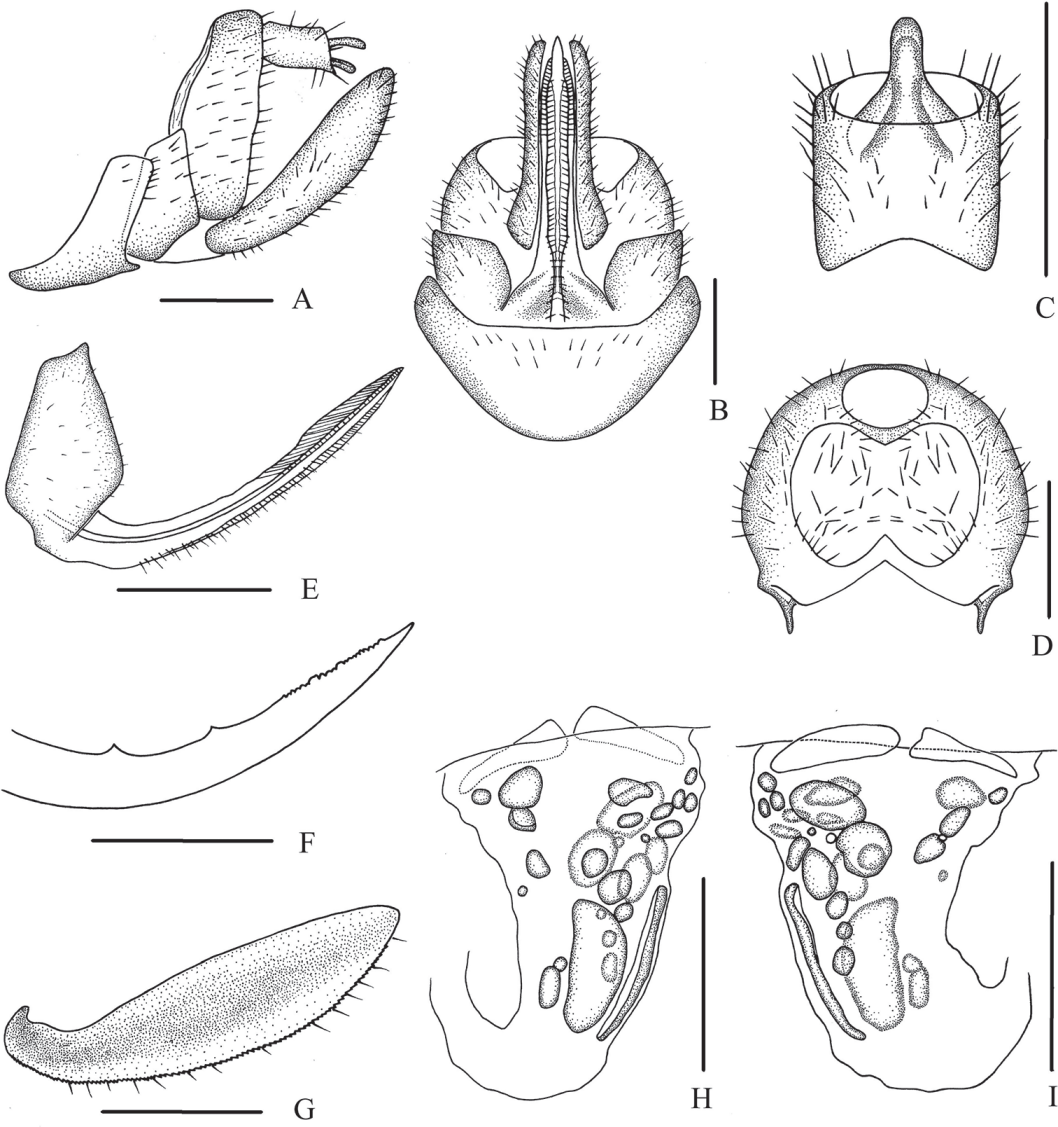
*Betacixius maguanensis* sp. nov., female **A** genitalia, lateral view **B** genitalia, ventral view **C** anal segment, dorsal view **D** tergite IX, caudal view **E** gonapophysis VIII and gonocoxa VIII, ventral view **F** gonapophysis IX, lateral view **G** gonoplac, inner lateral view **H** posterior vagina, ventral view **I** posterior vagina, dorsal view. Scale bars: 0.5 mm.

***Female genitalia*.** Tergite IX (Fig. 7A, B, D) moderately sclerotized, with a large, nearly dumbbell-shaped wax plate. Anal segment (Fig. 7C) rectangular, 1.1 times longer than wide in dorsal view, anal style finger-like. Gonapophysis VIII (Fig. 7E) elongate, and slightly curved upwards. Gonapophysis IX (Fig. 7F) with two middle teeth, distance ratio between distal middle tooth to apex and length of denticulate portion is 2.0. Gonoplac (Fig. 7G) rod-like, 3.8 times longer than wide in lateral view. Posterior vagina pattern as shown in Figure 7H, I.

#### Distribution.

China (Yunnan).

#### Etymology.

The species name is derived from Maguan County, Yunan Province, where the type locality is located.

#### Remarks.

Male genitalia of *B. maguanensis* sp. nov. is similar to *B. flagellihamus* Zhang & Chen, 2011, but differs in: (1) spinose process on right of periandrium nearly straight, directed cephalad (in *B. flagellihamus*, spinose process on right of periandrium curving dorsally); (2) apical lobes of anal segment pointed ventrally (the latter rounded); (3) forewing without ocellate marking (the latter with a large ocellate marking in apical half).

### Betacixius sparsus

Taxon classificationAnimaliaHemipteraCixiidae

Tsaur & Hsu, 1991

73715476-C183-58E1-806F-8A45E7F5915D

Figure 8A–I

Betacixius sparsus Tsaur and Hsu in Tsaur et al. 1991: 46, fig. 23A–E.

#### Material examined.

**China**: 20♂♂ 56♀♀, Guangxi Province, Wuming County, Daming Mountain (23°27'N, 108°27'E), 14–15 May 2012, Zhi-Hua Fan, Hu Li, Nan-nan Yang; 11♂♂ 11♀♀, Guangxi Province, Wuming County, Daming Mountain, 14–17 May 2011, Xiao-Fei Yu, Rong Huang, Xin-Feng Zhang; 4♂♂ 4♀♀, Guangxi Province, Shangsi County, Shiwandashan National Forest Park (21°56'N, 108°6'E), 2–4 May 2011, Xiao-Fei Yu, Rong Huang.

#### Supplementary description.

**Figure 8.**
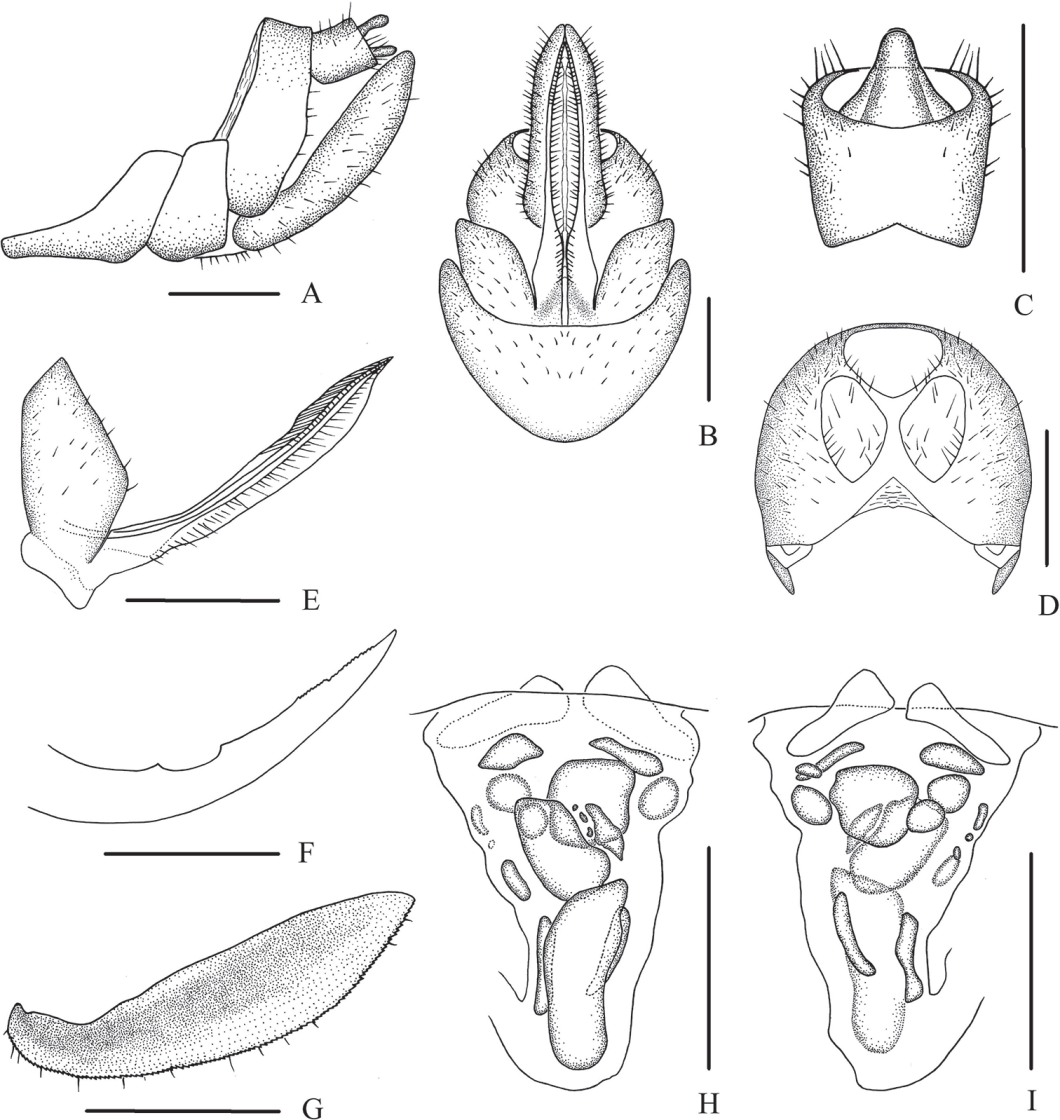
*Betacixius sparsus* Tsaur & Hsu, 1991, female **A** genitalia, lateral view **B** genitalia, ventral view **C** anal segment, dorsal view **D** tergite IX, caudal view **E** gonapophysis VIII and gonocoxa VIII, ventral view **F** gonapophysis IX, lateral view **G** gonoplac, inner lateral view **H** posterior vagina, ventral view **I** posterior vagina, dorsal view. Scale bars: 0.5 mm.

***Female genitalia*.** Tergite IX (Fig. 8A, B, D) moderately sclerotized, with two nearly oval wax plates, separated from each other. Anal segment (Fig. 8C) rectangular, widening to apex, 1.1 times wider than long in dorsal view, anal style strap-like. Gonapophysis VIII (Fig. 8E) elongate, and slightly curved upwards. Gonapophysis IX (Fig. 8F) with two middle teeth, distance ratio between distal middle tooth to apex and length of denticulate portion is 2.3. Gonoplac (Fig. 8G) rod-like, 3.8 times longer than wide in lateral view. Posterior vagina pattern as shown in Figure 8H, I.

#### Distribution.

China (Guangxi, Taiwan).

#### Note.

The female genitalia of this species are described and illustrated for the first time.

## Discussion

Prior to this study, nothing has been reported on the host plants of *Betacixius*. Nonetheless, we found these planthoppers most commonly in grass, shrubs, or on bamboo and tree leaves, at elevations up to 2600 m. Unfortunately, there is no knowledge of what plants these planthoppers really feed on, except that *B. flagellihamus* was collected on *Quercus* sp. (Fagaceae), which might be the plant on which it feeds.

**Figure 9.**
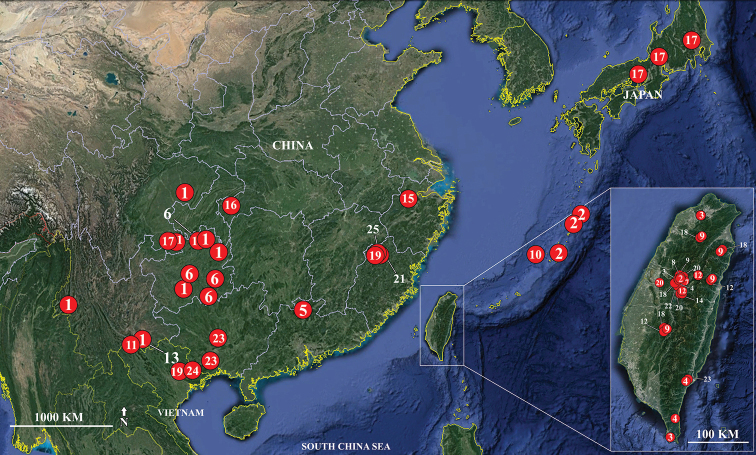
Distribution records of the species from genus *Betacixius***1***B. bispinus* Zhang & Chen, 2011 **2***B. brunneus* Matsumura, 1914 **3***B. clypealis* Matsumura, 1914 **4***B. delicatus* Tsaur & Hsu, 1991 **5***B. euterpe* Fennah, 1956 **6***B. flagellihamus* Zhang & Chen, 2011 **7***B. flavovittatus* Hori, 1982 **8***B. fuscus* Tsaur & Hsu, 1991 **9***B. herbaceus* Tsaur & Hsu, 1991 **10***B. kumejimae* Matsumura, 1914 **11***B. latissimus* sp. nov. **12***B. maculosus* Tsaur & Hsu, 1991 **13***B. maguanensis* sp. nov. **14***B. michioi* Hori, 1982 **15***B. nelides* Fennah, 1956 **16***B. nigromarginalis* Fennah, 1956 **17***B. obliquus* Matsumura, 1914 **18***B. ocellatus* Matsumura, 1914 **19***B. pallidior* Jacobi, 1944 **20***B. rinkihonis* Matsumura, 1914 **21***B. robustus* Jacobi, 1944 **22***B. shirozui* Hori, 1982 **23***B. sparsus* Tsaur & Hsu, 1991 **24***B. tonkinensis* Matsumura, 1914 **25***B. transversus* Jacobi, 1944.

Based on published data and our field surveys, the distribution of *Betacixius* is mostly restricted to the Oriental parts of China, Japan, and Vietnam, with *B. obliquus* Matsumura, 1914, occurring in both Palaearctic and Oriental regions, as the only exception (Fig. 9).

The regional studies on *Betacixius* in China are not equally efficient at finding species. To date, 23 species are known from China, of which about half that number (12 species) are known from Taiwan and only 12 species have been described from southern mainland China, which is much broader and more variant in ecological complexity compared with Taiwan. Therefore, we believe that additional comprehensive field surveys will find that the diversity of *Betacixius* in mainland China is doubtlessly richer.

## Supplementary Material

XML Treatment for Betacixius

XML Treatment for Betacixius bispinus

XML Treatment for Betacixius flagellihamus

XML Treatment for Betacixius latissimus

XML Treatment for Betacixius maculosus

XML Treatment for Betacixius maguanensis

XML Treatment for Betacixius sparsus

## Footnotes

revised from Fennah 1956; Zhang and Chen 2011
